# Developing High-Coloring Natural Systems Using Double Emulsions with *Daucus carota* L. Extract to Meet High-Performance Requirements

**DOI:** 10.3390/foods13244147

**Published:** 2024-12-21

**Authors:** Liandra Gracher-Teixeira, Samara C. Silva Pituco, Giovana Colucci, Arantzazu Santamaria-Echart, António M. Peres, Madalena M. Dias, M. Filomena Barreiro

**Affiliations:** 1 CIMO, LA SusTEC, Instituto Politécnico de Bragança, Campus de Santa Apolónia, 5300-253 Bragança, Portugal; liandra@ipb.pt (L.G.-T.); samaras@ipb.pt (S.C.S.P.); giovana.colucci@ipb.pt (G.C.); asantamaria@ipb.pt (A.S.-E.); peres@ipb.pt (A.M.P.); 2 LSRE-LCM—Laboratory of Separation and Reaction Engineering—Laboratory of Catalysis and Materials, Faculty of Engineering, University of Porto, Rua Dr. Roberto Frias, 4200-465 Porto, Portugal; dias@fe.up.pt; 3 ALiCE—Associate Laboratory in Chemical Engineering, Faculty of Engineering, University of Porto, Rua Dr. Roberto Frias, 4200-465 Porto, Portugal

**Keywords:** anthocyanins, black carrot (*Daucus carota* L.), natural colorant, double emulsion, coloring power, emulsion stability

## Abstract

*Daucus carota* L. extract is attracting interest as a natural colorant alternative. However, the presence of anthocyanins (ACNs), which are sensitive to pH changes, limits its application. To tackle this issue, water-in-oil-in-water (W_1_/O/W_2_) double emulsions are emerging as innovative solutions. Nevertheless, the problem of reaching robust colorant systems for industrial use still needs to be overcome. One important target is to reach a high coloring power, minimizing its impact on the final product. In this context, the effect of colorant concentration and the volume of the primary emulsion, two routes to increase the colorant power, on color attributes and stability, an important feature to reach a marketable product, was studied. The optimal experimental design was conducted to two optimal solutions, whether through heightened colorant concentration or primary emulsion volume: a 41/59 (W_1_/O)/W_2_ ratio with 11 wt.% colorant, and a 48/52 (W_1_/O)/W_2_ ratio with 6 wt.% colorant, respectively. A subsequent assessment of color and physical emulsion stability over 30 days pointed out the solution with the lower colorant concentration (6 wt.%) as the one with better performance (L*: 44.11 ± 0.03, a*: 25.79 ± 0.01, D_4;3_: 9.62 ± 0.1 µm, and CI: 14.55 ± 0.99%), also minimizing the permeability of the colorant to the outer aqueous phase. Overall, these optimized emulsions offer versatile coloring solutions suitable for various industrial applications, such as food matrices and functional cosmetics.

## 1. Introduction

Anthocyanins (ACNs) are water-soluble bioactive flavonoids found in flowers, roots, leaves, seeds, and stalks. ACNs cover a wide palette of colors, from blue and purple through orange to red [1,2]. They are used in the food industry (food colorant with E163 code), namely, in fruit juice concentrates, nectars, jellies, yogurts, marmalades, potato chips, ice creams, and soft drinks, replacing the synthetic Red 40 colorants [3]. They also have diversified pharmaceutical and cosmetic uses due to their coloring and antioxidant attributes [4,5]. Among ACN sources, black carrots (*Daucus carota* L.) are gaining interest due to their attractive color. They possess high levels of anthocyanins, mainly acylated cyanidin-3-(p-coumaroyl)-diglucoside-5-glucoside [6,7]. Black carrots are predominantly cultivated in Turkey, Afghanistan, Egypt, Pakistan, and India [1,7].

ACN stability is influenced by external factors, such as light, temperature, and pH, resulting in a loss of color and biological activity [5,8,9]. Moreover, ACN colors strongly vary with pH, from purple at a neutral pH to blue as pH rises. At a low pH, the flavylium cation is the predominant form characterized by a high water solubility and a strong red color. As pH rises, a colorless carbinol pseudo base and chalcone structures are formed, followed by anionic quinoidal species, resulting in a low red coloration at pH 4–5 [10,11,12]. Maintaining ACNs in a pH environment below four is important to maintain their strong red hue.

The stability of ACNs can be enhanced through protection in emulsion systems, namely, in water-in-oil-in-water ((W_1_/O)/W_2_) double emulsions. In these systems, the ACNs are entrapped in the internal aqueous phase (W_1_), which is dispersed as small droplets in the oil phase. The oil phase separates the internal aqueous phase from the external one (W_2_), protecting the ACNs against environmental changes [13,14]. Double emulsions can enhance foods’ nutritional, preservative, or protective qualities [15]. They are becoming increasingly popular in the food industry because of their resistance to environmental stresses, which makes them ideal for preserving and stabilizing various natural colorants [16].

Liu et al. (2019) [17] explored the efficacy of (W_1_/O)/W_2_ double emulsions in stabilizing black carrot ACNs against pH-induced color changes, focusing on their retention under different pH conditions (3 and 7) and storage temperatures (refrigerated at 4 °C and room temperature at 20 °C) for 7 days. Their results highlighted that anthocyanin leakage from the internal to the external aqueous phase was influenced by temperature, with a faster transference at room temperature due to ACNs’ higher solubility and diffusion to the oil phase. At a pH of 3, ACNs demonstrated higher retention within the W_1_/O droplets, suggesting that the solubility in the oil phase was reduced, likely due to the flavylium cation’s higher polarity at this pH. This behavior is typical of anthocyanins with present log P (partition coefficient) values close to or below zero. For example, for cyanidin-3-O-glucoside, a value of 0.39 was reported [18]. For the main back carrot compound, cyanidin-3-(p-coumaroyl)-diglucoside-5-glucoside, a value of −2.85 was predicted by Akinnusi and co-authors [19], indicating a polar nature and, thus, a preferential partitioning into the aqueous phase [20].

Sebben et al. (2021) [21], who tested different electrolyte compositions (adipic acid, potassium chloride, and citrate buffer) as the internal phases in (W_1_/O)/W_2_ emulsions for ACNs encapsulation, corroborated the advantages of this strategy. It helped to control the osmotic pressure gradient, ensuring the emulsion’s long-term physical stability while preserving the ACNs’ color and biological properties. In a previous study that we conducted, (W_1_/O)/W_2_ double emulsions were also tested as protective carriers for *Sambucus nigra* L. coloring extracts, minimizing their common color variability under pH changes, even though a low coloring power was noticed [22]. In addition to stability, achieving a suitable coloring power for ACN-based systems is of chief importance for industrial applications.

In the aforementioned context, this study targets the development of colorant systems based on (W_1_/O)/W_2_ double emulsions loaded with *Daucus carota* L. extract, focusing on the obtainment of robust coloring systems, i.e., products holding high-coloring power and physical stability. For that, a Central Composite Rotatable Design (CCRD) 2^2^ was applied to systematize the analysis of the extract concentration in the inner aqueous phase (W_1_) and the primary emulsion to the second aqueous phase ((W_1_/O)/W_2_) ratio on the emulsion coloring power and stability. Even though the existence of some studies points out the advantages of double emulsions in protecting ACN color against pH changes and their color intensification under low acidic pH, to the author’s best knowledge, this is the first-in-kind study where the colorant power was targeted, coupled with stability. This is a step forward to achieve high-performance solutions for industrial applications.

## 2. Materials and Methods

### 2.1. Samples and Reagents

The dried black carrot (*Daucus carota* L.) extract, supplied in powder form, was kindly donated by Secna Natural Colors, Spain, now operating as OTERRA. The extract was determined to contain 36 mg of anthocyanins per gram using the pH differential method. Samples were stored in refrigerated conditions (4 °C) and protected from light. Corn oil (Fula, Portuguese brand) was acquired from a local supermarket in Bragança, Portugal. Polyglycerol polyricinoleate (PGPR), a hydrophobic emulsifier (HLB of 1.5), was obtained from Palsgaard (Juelsminde, Denmark). Polysorbate 80 (Tween 80, HLB of 15) was obtained from Alfa Aesar (Karlsruhe, Germany). Gum Arabic, from the acacia tree, was purchased from Sigma (St. Louis, MO, USA). The citric acid was provided by Merck KGaA (Darmstadt, Germany). The used water was distilled water.

### 2.2. Experimental Design

To evaluate and optimize the color power and stability of the emulsion, a Central Composite Rotatable Design (CCRD) was carried out with two independent variables, the (W_1_/O)/W_2_ ratio (*x*_1_, *v*/*v*) and colorant concentration (*x*_2_, wt %). These variables were selected as relevant since the coloring power depends on the used colorant concentration and the used (W_1_/O)/W_2_ ratio. Based on a previous study that we conducted [22], the primary water-in-oil emulsion (W_1_/O) ratio was fixed as 40/60 (*v*/*v*), and the (W_1_/O)/W_2_ ratio changed between 20/80 and 50/50 (*v*/*v*). Preliminary experiments aimed at determining the optimal colorant concentration revealed a suitable range spanning from 0.8% (wt.%) as a minimum to 12% (wt.%) as a maximum. Concentrations exceeding this value resulted in incomplete dissolution. The CCRD included 3 replicates at the central point and 4 axial points (2^2^ + star configuration), resulting in 11 experimental runs. Second-order models for the predicted responses (Y; double emulsion volume-mean droplet diameter (D_4,3_), creaming index (CI), and color parameters (L* for lightness and a* for reddish)) were developed as a function of the defined variables (*x*_1_ and *x*_2_). Note that b* values were not considered due to the presence of both negative and positive values, rendering a comprehensive statistical analysis unfeasible. Nonetheless, a* and L* proved to be the most significant color parameters, as they are directly related to the pinkish (reddish) hue and the intensity of lightness/darkness (L*), respectively. These responses were modeled according to the general polynomial equation expressed in Equation (1).

(1) $$Y_{k}=a_{0,k}+\sum_{i=1}^{2}{(a}_{i,k}x_{i})+\sum_{i<j}^{2}{(a}_{ij,k}x_{i}x_{j}),with\;k=1\;to\;4\;$$

where $Y_{k}$ is the predicted response for the *k* parameter (i.e., D_4,3_; CI; a*; and L*), the $a_{i}$ and $a_{ij}$ coefficients are, respectively, the first- and second-order parameters whose values are determined using multiple linear regression models, and $x_{1}\;and\;x_{2}\;$ are the two actual independent factors of the (W_1_/O)/W_2_ ratio and colorant concentration, respectively).

The specified range of the independent variables is described in Table 1. Only one double emulsion was prepared per trial. The repeatability of the process was evaluated by using the 3 replicates of the central point, according to the applied CCRD method.

Second-order models were obtained and evaluated statistically using the software Design-Expert 6.0.6. (StatEase, Minneapolis, MN, USA), the trial version for all the data results. Analysis of Variance (ANOVA) CCRD 2^2^ (at a 5% significant level, i.e., *p*-value < 0.05) and the coefficient of determination (R^2^) were used to determine the adequacy of the fitted models. The regression fit was evaluated using the multiple and adjusted coefficients of determination (R^2^ or R^2^_adj_) and the predicted coefficient of determination (R^2^_pred_). The accuracy of the mathematical model in reproducing experimental data was checked by the R^2^ and R^2^_adj_ values. The predictive performance of the mathematical model regarding additional experiments can be inferred based on the R^2^_pred_. A satisfactory fit is ensured when R^2^ and R^2^_adj_ values are greater than 0.75. A good predictive capability requires a R^2^_pred_ higher than 0.60. A model is considered useless if the values are below 0.25.

The experimental runs were carried out randomly. During the model validation, the *t*-Student’s test was used to investigate the statistically significant differences between means of the best conditions at a 95% confidence level (*p*-value ≤ 0.05).

The coefficient of variation (CV) was calculated using Equation (2):

(2) $$CV\left(\%\right)=\frac{standard\;deviation}{mean}\times 100$$

### 2.3. Double Emulsion Production

The generation of (W_1_/O)/W_2_ double emulsions comprised two sequential stages. Initially, a primary emulsion (W_1_/O) was prepared. Subsequently, this W_1_/O emulsion was emulsified into a secondary water phase (W_2_) to attain the (W_1_/O)/W_2_ double emulsion. For the W_1_/O primary emulsion, the aqueous phase was prepared by dissolving the extract in the water phase, then stirred at 600 rpm using a magnetic stirrer (Rslab-1C, RSLab, Heraklion, Greece) for 15 min at room temperature. In the present study, the inner aqueous phase was acidified with citric acid (pH of 3) to potentiate color intensity. The oil phase was formulated by incorporating PGPR at 5% (wt.%, based on the oil) and then stirred under the conditions described earlier. The primary emulsion was then prepared by mixing the two phases at a W_1_/O ratio of 40/60 (*v*/*v*), using an Ultra-Turrax (Unidrive X1000 Homogenizer Drive—CAT Scientific, Staufen, Germany) under 20,000 rpm for 5 min. To produce the (W_1_/O)/W_2_ double emulsion, the external aqueous phase (W_2_) was prepared with water added with 15% (wt.%) gum Arabic. The homogenization of the W_1_/O with the W_2_ to prepare the double emulsion was carried out at 6000 rpm for 2 min using an Ultra-Turrax. Various (W_1_/O)/W_2_ (*v*/*v*) ratios and colorant concentrations (wt.%) were used according to the description of the 11 runs (E1-E11) of the CCRD 22 design matrix (Table 2).

### 2.4. Emulsion Characterization

#### 2.4.1. Droplet Size Analysis

Volume-mean droplet sizes (D_4,3_, µm) of the prepared emulsions were determined in a Malvern Mastersizer 3000 equipped with a Hydro MV dispersion unit (Malvern, UK), using water as the dispersing medium. The absorption was adjusted to 0.01, and the refractive index for the dispersed phase (corn oil) and continuous phase (water) were 1.48 and 1.45, respectively. Five consecutive measurements were performed for each sample. The retrieved data were treated using the Malvern Software version 3.10. Sampling times of 1, 7, 15, and 30 days were considered.

#### 2.4.2. Creaming Index Evaluation

To determine the CI, the double emulsion was transferred into clear cylindric glass jars of 27 mm × 60 mm in dimension and stored at 4 °C since color stability is favored under refrigerated conditions [22]. Moreover, this temperature is supported by the potential applications of the coloring systems in refrigerated food products, such as yogurts and other chilled products. Through the CI determination, it is possible to assess, macroscopically, the emulsion stability. At each measurement time (1, 7, 15, and 30 days), the jars were carefully removed from the refrigerator to preserve the integrity of the emulsion layers, and photographs were taken to document their visual appearance. In addition, the height of the upper distinctive serum layer (h_s_) and the total height of the emulsion (h_t_) were measured in centimeters. These values were then used in the following Equation (3) to calculate CI (%):

(3) $$CI\left(\%\right)=\frac{h_{s}}{h_{t}}\times 100$$

#### 2.4.3. Emulsion Microstructure

The emulsion microstructure was examined by optical microscopy, which is a suitable technique to perceive early signs of instability. The used apparatus was a Nikon Eclipse 50i microscope (Tokyo, Japan) equipped with a Nikon Digital camera and NIS-Elements Documentation software version 5.01. For the analysis, an aliquot of the emulsion was placed on a slide and gently covered with a coverslip. The emulsions were observed using sampling times of 1, 7, 15, and 30 days, similar to the creaming index evaluation.

#### 2.4.4. Color Determination

The color of the emulsions was determined using a Konika Minolta Sensing Inc. CR-400 colorimeter. It provides the determination of L* (lightness), a* (green to red), and b* (blue to yellow) parameters. Only the emulsified layer was used in these measurements. Regarding coloring power, for the studied samples, higher a* values indicate a more pronounced red color, whereas lower L* values indicate a darker shade. Low changes in both a* and L* indicate the color stability of the emulsion. To assess color stability over time, measurements were conducted at 1, 7, 15, and 30 days. Each sample was measured in triplicate.

## 3. Results and Discussion

### 3.1. Emulsion Characterization

**Figure 1:**
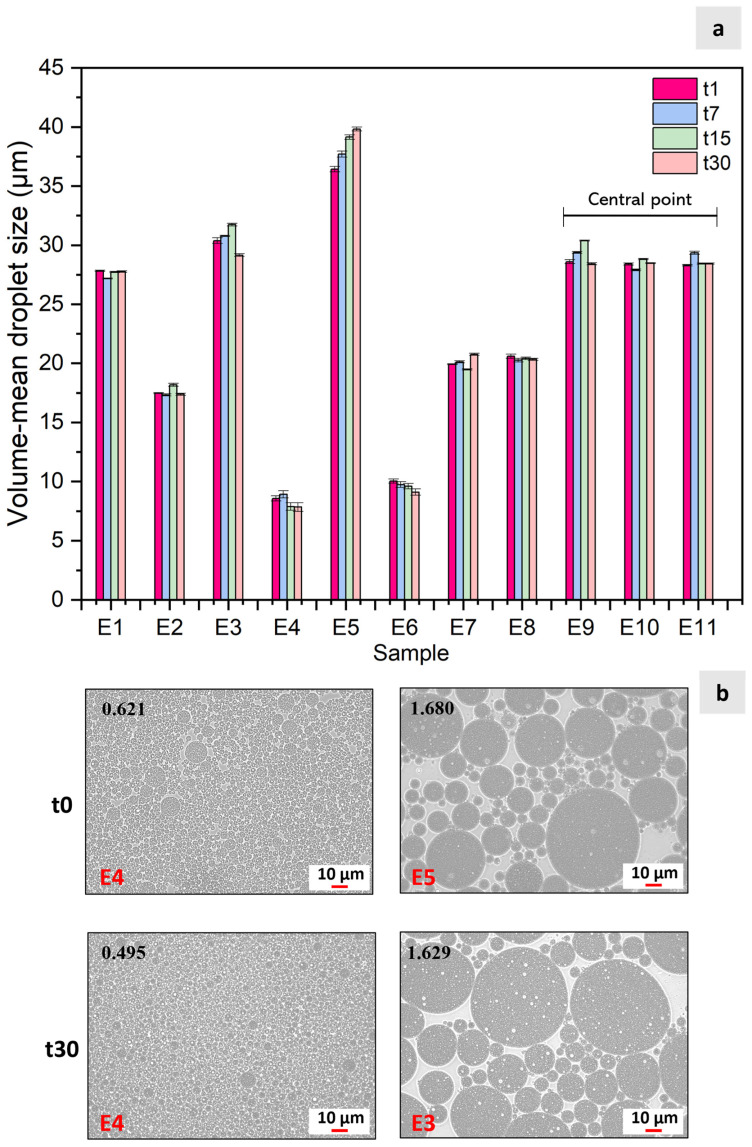
(**a**) Results of the 11 experimental runs (emulsions) obtained by the Central Composite Rotatable Design 2^2^ for D_4,3_ over time: 1 day (t1); 7 days (t7); 15 days (t15); and 30 days (t30); (**b**) Microscopy images comparing the samples with smaller (E4) and larger (E5 and E3) droplet sizes at time 0 and after 30 days. The Span values, indicating sample size homogeneity, are displayed in bold in the upper left corner.

Figure 1a presents the size (D_4,3_) evolution over time for the emulsions prepared according to the experimental design (E1–E11), as shown in Table 2 of the Section 2. The complete data are displayed in Table S1 (Supplementary Materials), which presents the design matrix of the CCRD with the independent variables (emulsion ratio (*v*/*v*) and colorant concentration (%)), along with the responses in the volume-mean droplet size (µm) and the creaming index (%), measured at 1, 7, 15, and 30 days. The size of the oil droplets of the double emulsion ranged from 8.67 ± 0.22 μm to 36.60 ± 0.23 μm, which were obtained for the samples (E4, 46/54/10.37) and E5 (20/80/6.40), respectively. After 30 days (t30), this trend was maintained, with E4 achieving the lower size (7.84 ± 0.37 μm) and E5 the higher one (39.82 ± 0.16 μm). Concerning the central point (E9, E10, and E11; 35/65/6.40), similar values for the droplet size with coefficients of variation between 0.1 and 3.5% were achieved, demonstrating satisfactory precision and repeatability. In general, for all runs, the D_4,3_ did not change considerably with time, indicating better stability than other reported systems. For example, Li et al. (2023) [23] reported that, in their double emulsion system to protect anthocyanins, the average droplet size increased from 28.78 μm to 37.50 μm and 45.62 μm after 28 days of storage at 4 °C and 25 °C, respectively. Similarly, Teixé-Roig et al. (2018) [24] reported that double emulsions stabilized by PGPR and lecithin increased from 6 μm to 24 μm after 21 days of storage at 4 °C.

Concerning droplet size, high Span values align with wide size distributions (higher size heterogeneity), whereas lower values denote narrow distributions (higher size homogeneity [25]. In this context, E4 (46/54/10.37) exhibited a lower Span value (0.621), whereas the higher one was found for E5 (20/80/6.40). For 30 days, the lower value was also achieved for sample E4 (0.495), whereas the higher one was achieved for E3 (1.629). These observations were corroborated by the microscopic analysis, as shown in Figure 1b. The complete morphological data (t1, t7, t15, and t30) can be found in Table S2 of the Supplementary Materials, where images of all 11 samples from the CCDR 2^2^ experimental runs are presented. Moreover, by optical microscopy (Figure 1b), it can be perceived that each large droplet contains smaller droplets, producing a “droplet in droplet” structure in accordance with a double emulsion structure [26].

**Figure 2:**
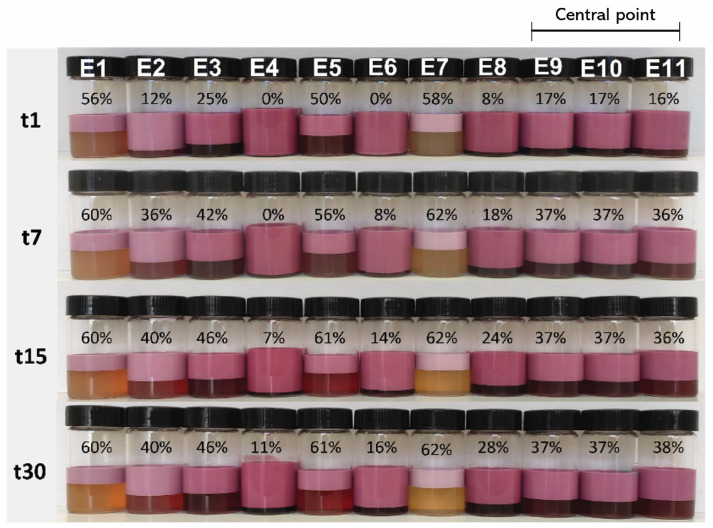
Results of the 11 experimental runs (emulsions) obtained by the Central Composite Rotatable Design 2^2^ for the Creaming Index (CI) % over time: 1 day (t1); 7 days (t7); 15 days (t15); and 30 days (t30).

The creaming index (CI) values of the double emulsions over the 30-day analysis period are depicted in Figure 2. In general, CI values increased over time, indicating a decrease in emulsion stability. The E4 (46/54/10.37) sample remained the most stable throughout the study period, with a CI consistently below 11%. Conversely, E5 (20/80/6.40) and E7 (35/65/0.80) showed higher CI values, reaching 61% and 62%, respectively, after 30 days. Similarly, Parralejo-Sanz et al. (2024) [27] observed that, in their double emulsions used to entrap betalains, phase separation started to be perceptible after four days at 7 °C and increased five-fold over the assayed 20 days. Teixeira et al. (2022) [22] also described an increase in the phase separation; when a 20/80 (W_1_/O)/W_2_ ratio was used, a CI of 68% was reached on the first day, increasing to 74% after 20 days. In contrast, when the ratio was 50/50, the CI was 0 on the first day and then increased to 20% after 20 days.

These findings are consistent with the results of the D_4,3_, corroborating the influence of droplet size on emulsion stability [28,29]. Additionally, E9, E10, and E11, identified as central points (replicas), exhibited a consistent trend in the CI evolution over time, with coefficients of variation ranging from 0 to 3.8%, indicating good precision and repeatability of the assays.

**Figure 3:**
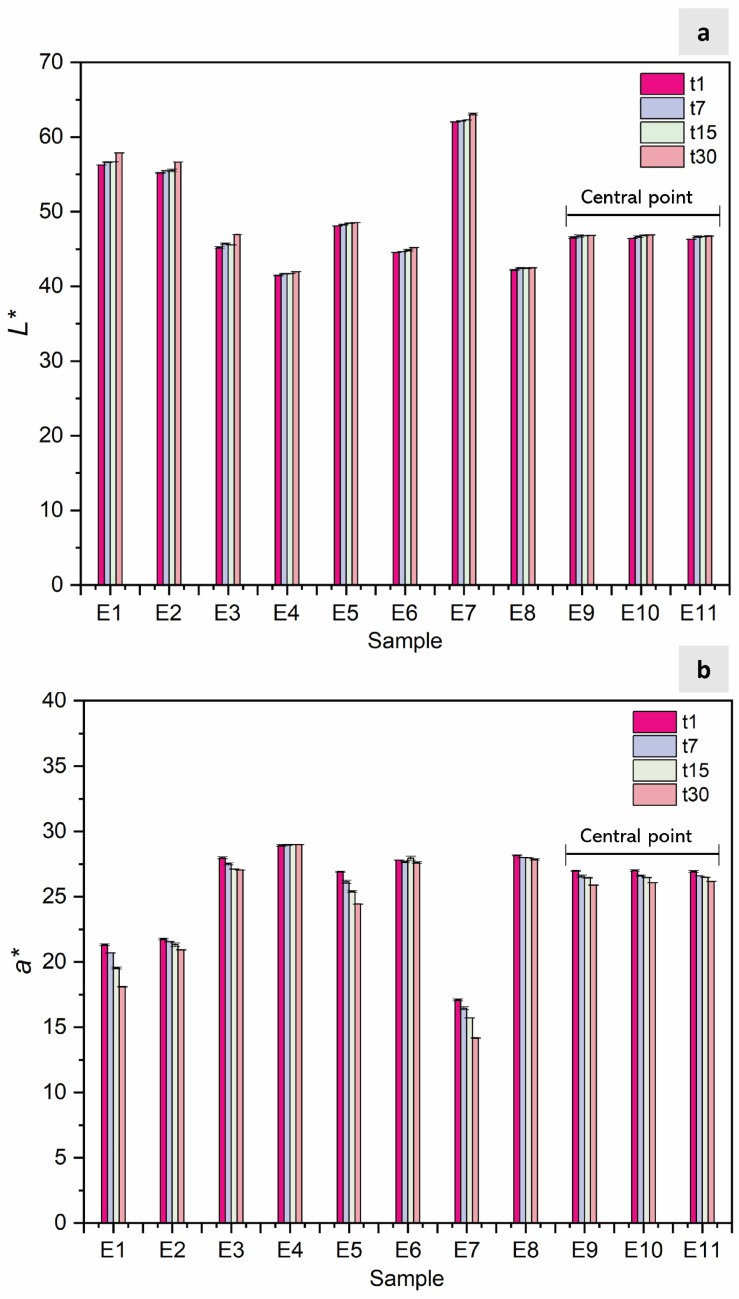
(**a**) Results of the 11 runs (emulsions) obtained by the Central Composite Rotatable Design 2^2^ for colorimetric parameters: L* (**b**) and a* over time: 1 day (t1); 7 days (t7); 15 days (t15); and 30 days (t30).

In Figure 3a,b, the color parameters L* and a* are depicted over the 30-day analysis period, with complete data available in Table S3 (Supplementary Materials). Table S3 includes the design matrix of the CCRD with independent variables (emulsion ratio (*v*/*v*) and colorant concentration (%)) and the colorimetric response (parameters a* and L*) for 1, 7, 15, and 30 days. Parameter a* consistently reached positive values, indicating samples with reddish tones. E7 (35/65/0.80) exhibited the lowest a* values and highest L* values throughout this study (t1: a* = 17.09 ± 0.06, L* = 62.01 ± 0.04; t30: a* = 14.17 ± 0.05, L* = 63.07 ± 0.13), indicating a lower colorant power, while E4 (46/54/10.37) was characterized by the highest a* values and lowest L* values (t1: a* = 28.92 ± 0.07, L* = 41.44 ± 0.05; t30: a* = 28.98 ± 0.01, L* = 41.95 ± 0.02), indicating higher colorant power with minimal changes over time. For E9, E10, and E11 (replicas of the central point), similar values were observed over time, with a CV varying from 0.15 to 0.54% for a* and between 0.1 and 0.2% for L*. Li and coworkers [23] observed in their (W_1_/O)/W_2_ system, which was used to entrap anthocyanins from *Nicandra physalodes* (Linn.) Gaertn. seeds that L* significantly increased (from 62 to 71) and a* decreased (from 15 to 9) over 28 days in a dark environment, indicating that the red color gradually faded. Similarly, Eisinaitė et al. (2020) [30] observed a significant loss of the red color (a* decreased from 21.83 ± 0.22 at time 0 to 17.02 ± 0.13 after 30 days), with L* values remaining relatively constant during the first 30 days (from 54.83 ± 0.54 to 56.22 ± 0.30). These results suggest that the studied system maintains a more consistent color over more extended periods.

### 3.2. Statistical Analysis

Mathematical models were fitted to the experimental data to allow a more consistent description of the effect of the colorant concentration and the (W_1_/O)/W_2_ ratio combinations. The adequacy of the fitted models was verified using ANOVA. The results for the t1 period (Table 3) showed that it was possible to establish a multivariate significant model (*p*-value ≤ 0.0002) for each of the four dependent studied variables (D_4,3_, CI, a* and L*). Each model included only the factors (linear, interaction, and/or quadratic order) for which a significant effect was observed (*p*-value ≤ 0.05 or *p*-value ≤ 0.10). In some cases, non-significant lower-order factors were included to ensure that the model is hierarchical (e.g., the inclusion of the non-significant linear *x_2_* in the D_4,3_ model). The R^2^ and R^2^_adj_ values ranged between 0.9069 and 0.9964, which are greater than 0.75, showing a good goodness of fit. In addition, the R^2^_pred_ (varying from 0.7893 and 0.9826) are generally in reasonable agreement with the R^2^_adj_ (similar magnitude), the former values greater than 0.60 [31,32,33], demonstrating satisfactory goodness of prediction, allowing the use of the models to predict new experiments. The central points (E9, E10, and E11) showed slight variations along the results, corroborating the satisfactory repeatability of the process for all studied responses [34,35].

**Figure 4:**
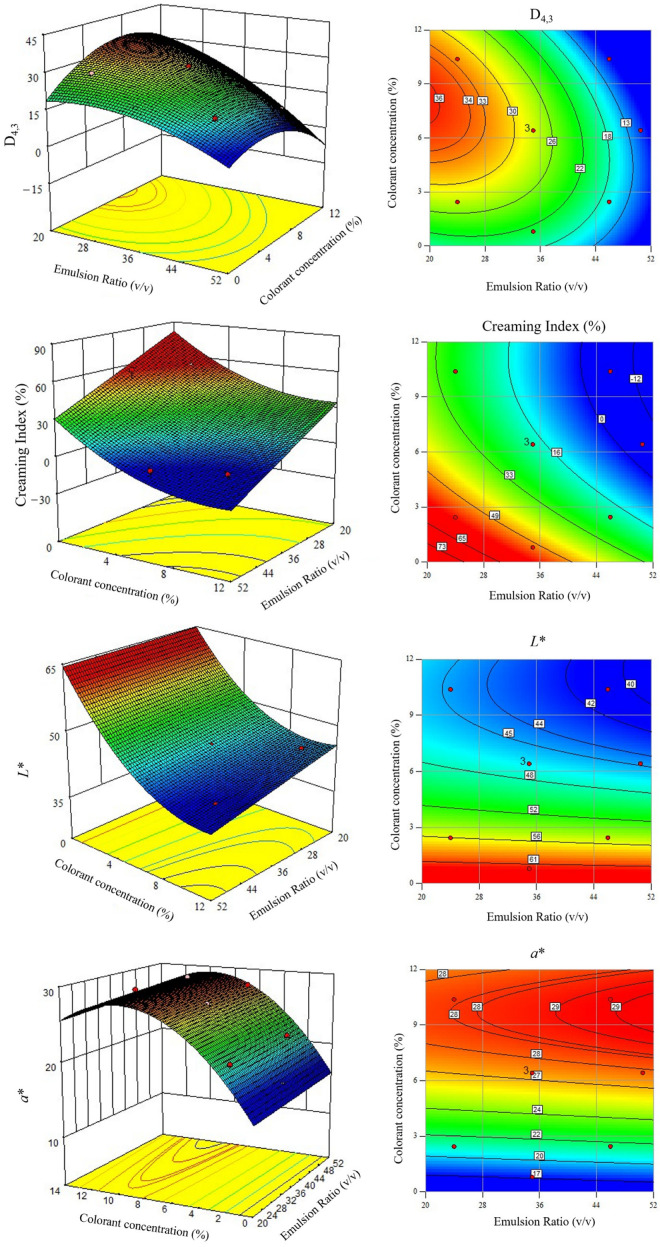
Response surfaces for the volume-mean droplet size (µm), the creaming index (%), and colorimetric parameters (L* and a*) as a function of colorant concentration (wt.%) and the emulsion ratio (*v*/*v*).

Table 3 shows the second-order models developed for the D_4,3_ (μm), CI (%), and colorimetric parameters (a* and L*) as a function of the (W_1_/O)/W_2_ ratio (*x_1_*) and colorant concentration (*x_2_*) for the first studied time (t1). The predictive models for D_4,3_, CI, and color parameters (L* and a*) were used to generate response surface graphics for t1, which are displayed in Figure 4.

The developed models (Table 3) were used to optimize the experimental conditions. As a first approach, a numerical optimization was performed to determine, within the studied experimental ranges of the (W_1_/O)/W_2_ ratio (*x*_1_) and colorant concentration (*x*_2_), the optimal conditions that would simultaneously minimize D_4,3_, minimize the CI, and maximize the a*. No optimization goal was established for L*, and the values were sought to remain within the experimental range. Three optimal solutions were obtained for the (W_1_/O)/W_2_ ratio of 46/54 or 43/57 and the colorant concentration ranging from 9.16 to 10.10%. However, based on the established models, a negative CI would be predicted (ranging from −5 to −2), which lacks physical meaning. Thus, alternatively, a graphical optimization approach was implemented based on the overlay plot of the developed models for D_4,3_, CI, and a*. For that, the following constraints were set to avoid selecting physically meaningless optimal solutions: D_4,3_ < 16 mm; 0 < CI < 10%; and a* > 26.5. The values were established based on the understanding that emulsions with smaller droplets generally exhibit greater stability, resulting in lower CI values [27].

This study’s primary objective was to achieve the highest a* value, indicating a more reddish hue, which was ensured by imposing an a* value greater than a minimum value. This approach is beneficial for reaching meaningful solutions for industrial use, as it allows for vibrant coloring without incorporating a large quantity of the double emulsion system into the final product. Although a model was developed for L*, this parameter was not considered in the optimization process as it did not constitute a critical condition for emulsion selection. In fact, the present study’s focus was mainly on achieving the smallest value, with no specific value pre-established.

**Figure 5:**
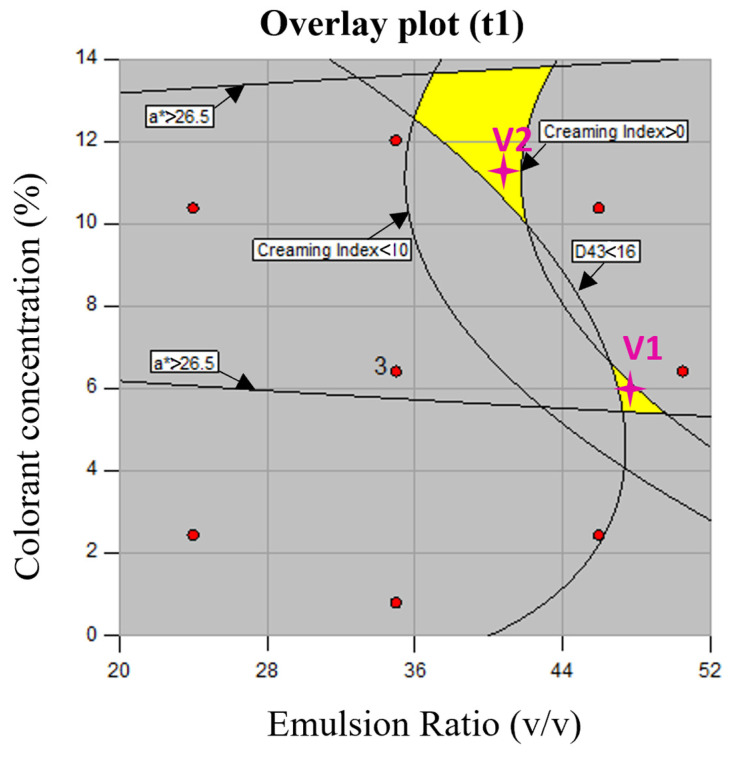
Overlay of the contour plots for D_4,3_, creaming index, a*, and L* depicting the optimum conditions (yellow area). The droplet size was set smaller than 16 µm, 0 < creaming index < 10%, a* > 26.5, and L* within the experimental range.

The overlay output for the initial time (t1) is shown in Figure 5. As can be inferred, two yellow color regions can be observed in the design space, corresponding to different optimal combinations of the two studied independent variables ((W_1_/O)/W_2_ ratio and colorant concentration) that fulfill the above-mentioned constraints. Thus, among the possible combinations, one optimal solution was used for each region, considering the main objective of obtaining an emulsion with a minimum D_4,3_ and CI. For the lower yellow optimal region, the following solution was graphically selected: a (W_1_/O)/W_2_ ratio of 48/52 and a colorant concentration of 6 wt.% (V1). For the upper yellow optimal region, the following conditions were chosen: a (W_1_/O)/W_2_ ratio of 41/59 and a colorant concentration of 11 wt.% (V2).

Based on the developed models and for the considered V1 optimal solution, the predicted minimum values of D_4,3_ and the CI were equal to 14.63 mm and 0.3%, respectively, and the maximum predicted value of a* was equal to 27.05. For this set, an optimal L* equal to 45.93 was predicted. For V2, the minimum predicted values of D_4,3_ and the CI were equal to 13.82 mm and 1.3%, and the maximum predicted a* equal to 28.50. In this case, a predicted L* value of 41.61 was calculated. Three experimental validation assays were conducted for each formulation (V1 and V2), and the experimental values of D_4,3_, the CI, a*, and L* were determined over a 30-day period. Throughout this time period, the recorded values for V1 ranged between 9.62 and 9.72 μm for D_4,3_, 0 and 14.55% for the CI, 26.13 and 25.79 for a*, and 43.80 and 44.11 for L*. For V2, the experimental values ranged between 17.63 and 18.25 μm for D_4,3_, 0 to 17.28% for the CI, 26.81 to 26.34 for a*, and 37.32 to 37.55 for L*.

The experimental values recorded for the V1 emulsion were similar to those predicted for a* and L* over the 30 days. However, the observed values for L* were lower than those predicted, which was positive from a practical point of view since the obtained emulsion was darker than the predicted one. Additionally, the experimental values for D_4,3_ were smaller than the predicted values, which were also positive results. In contrast, except for t1, it was observed that, during the storage period, the emulsion tended to acquire higher values of the CI than those predicted by the optimized model.

As for V2, the predicted a* and L* values were also of the same magnitude as the experimental ones, with higher values for a* and smaller values for L*, resulting in a darker sample than that predicted by the model. It should be remarked that, except for t1, for the V2 emulsion, the experimental D_4,3_ and CI values along the 30 days were higher than those predicted by the developed models. The experimental validation data, including the comparative stability results for the two optimal solutions, are presented in Table S4 (Supplementary Materials). This table provides an overview of the experimental values (D_4,3_, CI, L*, and a*) obtained for V1 (48/52 (W_1_/O)/W_2_ ratio (*v*/*v*), and 6 wt% colorant) and V2 (41/59 (W_1_/O)/W_2_ ratio (*v*/*v*) and 11 wt% colorant). Even if V2 allowed obtaining a darker emulsion in comparison with V1, unfortunately, the permeability of a fraction of colorant to the outer phase of the emulsion was observed, as can be visualized in Figure S1 (Supplementary Materials). This effect might be associated with the smaller droplet size observed for V1 (D_4,3_ ≈ 9.6 μm) compared to V2 (D_4,3_ ≈ 18 μm), contributing to better emulsion stability by minimizing gravitational separation and coalescence. The smaller droplet size helps maintain interfacial integrity and may reduce the likelihood of colorant migration between phases [36].

The effects of the emulsions’ storage time (1 to 30 days) with the two optimal compositions (V1 and V2) were further assessed using a two-way ANOVA, followed when appropriate by Tukey’s multi-comparison test. The results are shown in Table 4.

The two-way ANOVA highlights that the interaction effect (“emulsion composition × time”) was not statistically significant for the variables D_4,3_, CI, and a* (*p*-value > 0.05) allowing us to discuss each effect individually. Concerning L*, a significant interaction was found (*p*-value < 0.05). Thus, based on the results from Tukey’s multi-comparison test (Table 4), it is clear that, compared with V2, the emulsion V1 had significantly greater values of D_4,3_ and the CI and a lower a* value, pointing out that V1 is the emulsion with the best characteristics, namely, the lowest droplet size and lower creaming index, independently of the storage time. On the other hand, concerning the storage time, independently of the studied emulsion, the most evident effect was observed for the CI, which showed an undesirable significant increasing trend with the time, although a maximum value lower than 16% was observed.

To evaluate the trend of the optimal regions established from the CCRD (Figure 5) with the storage time, the CCRD was extended for 30 days, allowing us to establish models for the variables under study (D_4,3_, CI, a* and L*) after 7, 15, and 30 days (t7, t15, and t30). Tables S5–S7 (Supplementary Materials) list the second-order developed models, where the regression parameters (a’s coefficients of Equation (1)) for the volume-mean droplet size (μm), creaming index (%), and colorimetric parameters (a* and L*), and their second-order models, are included for 7, 15, and 30 days, respectively. The developed models enabled us to perform a graphical optimization, similar to what was previously performed for t1. The optimal regions (yellow color) under the same constraints (D_4,3_ < 16 mm; 0 < CI < 10%; and a* > 26.5) are shown in Figure S2 (Supplementary Materials). As can be visualized, the two initial optimal yellow areas found for t1 merged into a single area with time. The optimal region tended to be a region corresponding to the highest values for the (W_1_/O)/W_2_ ratio and concentration colorant, known to be unstable conditions for an emulsion. These results confirmed the goodness of the strategy used in the present study, based on the optimization using the initial time period. Even though the optimal regions identified for t15 and t30 (Figure S2) do not contain any of the previously established optimal solutions (V1 and V2 at t1), it is worth noting that these regions correspond to unstable emulsion formulations, as discussed earlier.

To overcome the curiosity, a test was performed using 12 wt.% of colorant concentration and a 50/50 (W_1_/O)/W_2_ ratio. Nevertheless, during the visual inspection analysis, it was observed that a fraction of the colorant permeated out immediately after emulsion production when a concentration of 12 wt.% was used, as observed in V2. This occurrence can be attributed to the high osmotic pressure resulting from a significant concentration gradient between the two aqueous phases. Considering the osmotic pressure difference between W_1_ and W_2_, W_1_ droplets have the potential to either shrink or swell, depending on the interaction with the W_2_ phase [37]. Due to this phenomenon, it was not feasible to implement the use of the highest colorant concentration.

## 4. Conclusions

Double emulsions are emerging as promising solutions to develop anthocyanin-based colorant systems due to their protective effect against pH changes. In this context, one important point is to reach effective colorant systems, namely, by targeting high colorant power and emulsion physical stability, which was the main focus of the present work. To accomplish this objective, a systematic study supported by a Central Composite Rotatable Design (CCRD) was conducted. After the identification of two optimal solutions, the evaluation of the target responses along time (volume-mean droplet size, creaming index, and colorimetric parameters) led to a best-performing formulation comprising a 48/52 (W_1_/O)/W_2_ ratio and 6 wt.% colorant content. It demonstrated robust and consistent coloring abilities, along with adequate physical stability, and was also able to prevent any leakage of the colorant into the outer aqueous phase. These attributes make it a promising candidate for use as a colorant in a range of foods like pastries, yogurt, beverages, and ice cream, where color stability is crucial for the final consumer. In addition, since anthocyanins have bioactive activities, the developed formulation can be valorized as a colorant and a functionalizing agent for foods, dermo-pharmaceuticals, and cosmetology industries. Applications could include products like lip gloss and foundations, providing vibrant and stable pigmentation.

## Figures and Tables

**Table 1:** Variable range applied in the Central Composite Rotatable Design 2^2^.

	Central Composite Rotatable Design 2^2^					
		−1.41	−1	0	+1	+1.41
Emulsion ratio (W_1_/O)/W_2_ (*v*/*v*)	$x_{1}$	20/80	24/76	35/65	46/54	50/50
Colorant concentration (wt.%)	$x_{2}$	0.80	2.43	6.40	10.37	12.00

**Table 2:** Central Composite Rotatable Design (CCRD) 2^2^ matrix with the coded levels and real values (in parentheses). *x_1_*: emulsion ratio ((W_1_/O)/W_2_) (*v*/*v*); *x_2_*: colorant concentration (wt.%).

Run	Independent Variables	
	Emulsion Ratio (W_1_/O)/W_2_ (*v*/*v*)	Colorant Concentration (wt.%)
	X_1_ (*x*_1_)	X_2_ (*x*_2_)
E1	−1 (24/76)	−1 (2.43)
E2	+1 (46/54)	−1 (2.43)
E3	−1 (24/76)	+1 (10.37)
E4	+1 (46/54)	+1 (10.37)
E5	−1.41 (20/80)	0 (6.40)
E6	+1.41(50/50)	0 (6.40)
E7	0 (35/65)	−1.41 (0.80)
E8	0 (35/65)	+1.41 (12.00)
E9	0 (35/65)	0 (6.40)
E10	0 (35/65)	0 (6.40)
E11	0 (35/65)	0 (6.40)

**Table 3:** Regression parameters (a’s coefficients of Equation (1)) for the volume-mean droplet size (μm), the creaming index (%) and colorimetric parameter (a* and L*) responses, and their second-order models for the first day (t1).

Source	Droplet Size (D_4,3_)		Creaming Index (CI)		L*		a*	
	a’s Coefficients ^#^ (Actual Factors)	*p*-Value	a’s Coefficients ^#^ (Actual Factors)	*p*-Value	a’s Coefficients ^#^ (Actual Factors)	*p*-Value	a’s Coefficients ^#^ (Actual Factors)	*p*-Value
Model	--	0.0001	--	0.0002	--	<0.0001	--	<0.0001
Intercept	+3.74	<0.0001	+113.4	<0.0001	+65.1	<0.0001	+14.4	<0.0001
*x*_1_—Emulsion Ratio	+1.21	<0.0001	−1.59	0.0001	−0.0135	0.0005	+0.0298	0.0209
*x*_2_—Colorant Concentration	+5.59	0.2744	−8.46	0.0005	−3.50	<0.0001	+2.74	<0.0001
*x_1_*_×2_	−0.0654	0.0128	--	n.s.	−0.0153	0.0371	--	n.s.
(*x*_1_)^2^	−0.0226	0.0077	--	n.s.	--	n.s.	--	n.s.
(*x_2_*)^2^	−0.270	0.0011	+0.380	0.0567	+0.185	<0.0001	−0.141	<0.0001
Quality Parameter	Values		Values		Values		Values	
Adequate Precision	23.38		16.10		61.31		60.73	
R^2^	0.9852		0.9348		0.9964		0.9951	
R^2^_adj_	0.9704		0.9069		0.9939		0.9931	
R^2^_pred_	0.8950		0.7893		0.9776		0.9826	

^#^ Parameter not statistically significant (n.s.; *p*-value ≥ 0.10): not included in the model since it did not affect its hierarchy.

**Table 4:** Results of each emulsion composition (emulsion ratio (*v*/*v*); colorant concentration (wt.%)). The results are presented as the mean ± standard deviation.

Effect		D_4,3_	CI	L*	a*
Emulsion composition	V1 (48/52, 6%)	9.85 ± 0.44 ^b^	7.12 ± 5.20 ^a^	44.00 ± 0.14 ^a^	26.01 ± 0.19
	V2 (41/59, 11%)	17.72 ± 0.53 ^a^	7.83 ± 6.30 ^a^	37.40 ± 012 ^b^	26.61 ± 0.27
	*p-*value	<0.0001	0.1025	<0.0001	<0.0001
Time	t1	13.68 ± 3.96 ^a^	0.00 ± 0.00 ^c^	40.56 ± 3.24 ^c^	26.47± 0.35 ^a^
	t7	13.84 ± 3.64 ^a^	6.24 ± 1.35 ^b^	40.64 ± 3.40 ^b,c^	26.51 ± 0.39 ^a^
	t15	13.93 ± 4.13 ^a^	7.72 ± 0.72 ^b^	40.76 ± 3.28 ^a,b^	26.11 ± 0.23 ^b^
	t30	13.70 ± 4.12 ^a^	15.92 ± 1.60 ^a^	40.83 ± 3.28 ^a^	26.14 ± 0.36 ^b^
	*p-*value	0.8275	<0.0001	0.0001	0.0007
Emulsion composition × Time	*p-*value	0.3320	0.1026	0.0184	0.5325

In each column and for each emulsion ratio and colorant, different letters mean significant differences for Tukey’s multi-comparison test (*p*-value < 0.05).

## Data Availability

The original contributions presented in this study are included in this article/Supplementary Materials, and further inquiries can be directed to the corresponding author.

## Supplementary Materials

The following supporting information can be downloaded at https://www.mdpi.com/article/10.3390/foods13244147/s1, Table S1: Design matrix of the Central Composite Rotatable Design (CCRD) with independent variables emulsion ratio (*v*/*v*) and colorant concentration (%) and the results for responses: Volume-mean droplet size (µm) and Creaming index (%) for the 1, 7, 15, and 30 days after the emulsion production; Table S2: Morphology of the 11 samples from the CCDR 2^2^ experimental runs. The Span values for t1 and t30 are represented in bold in the upper left side of the images; Table S3: Design matrix of the Central Composite Rotatable Design (CCRD) with independent variable emulsion ratio (*v*/*v*) and colorant concentration (%) and the results for responses: Colorimetric parameters: *a** and *L** for the 1, 7, 15, and 30 days after the emulsion production; Table S4: The experimental values of the validation formulations. V1: 48/52 (W_1_/O)/W_2_ ratio (*v*/*v*) and 6 wt% of colorant concentration. V2: a 41/59 (W_1_/O)/W_2_ ratio (*v*/*v*) and a colorant concentration of 11 wt.%; Table S5: Regression parameters (a’s coefficients of Equation (1)) for the volume-mean droplet size (μm), creaming index (%), and colorimetric parameters (*a** and *L**) responses, and their second-order models after seven days (t7); Table S6: Regression parameters (a’s coefficients of Equation (1)) for the volume-mean droplet size (μm), creaming index (%) and colorimetric parameters (*a** and *L**) responses, and their second-order models after fifteen days (t15); Table S7: Regression parameters (a’s coefficients of Equation (1)) for the volume-mean droplet size (μm), creaming index (%), and colorimetric parameters (*a** and *L**) responses, and their second-order models after third days (t30); Figure S1: The visual inspection of V2, where the fraction of colorant is in the outer phase of the emulsion; and Figure S2: The overlay plot for t1, t7, t15, and t30 after double emulsion production.
