# Supplementary material for: Developing High-Coloring Natural Systems Using Double Emulsions with Daucus carota L. Extract to Meet High-Performance Requirements
Source: Foods. 2024 Dec 21;13(24):4147. doi: 10.3390/foods13244147 (PMC11675280; doi:10.3390/foods13244147)
Supplement: Supplementary file 1 [file foods-13-04147-s001.zip › foods-3374214-supplementary.pdf]

Supplementary Material

Developing high-coloring natural systems using double emulsions with *Daucus carota* L. extract to meet high-performance requirements

Liandra Gracher-Teixeira [1,2,3], Samara C. Silva Pituco [1,2,3], Giovana Colucci [1,2,3], Arantzazu Santamaria-Echart [1] António Peres [1], Madalena M. Dias [2,3], and M. Filomena Barreiro [1,*]

Table S1: Design matrix of the Central Composite Rotatable Design (CCRD) with independent variables emulsion ratio (v/v) and colorant concentration (%) and the results for responses: Volume- mean droplet size (μm) and Creaming index (%) for the 1, 7, 15 and 30 days after the emulsion production.

	**Independent Variables**				**Dependent Variables**							
**Run**	**coded levels**		**real values**		**t1**	**t7**	**t15**	**t30**	**t1**	**t7**	**t15**	**t30**
	**x1**	**x2**	**Emulsion ratio ($(W_1/O)/W_2$) (v/v)**	**Colorant concentration (%)**	**Volume-mean droplet size – $D_{4.3}$ (μm)**				**Creaming Index (%)**			
E1	-1	-1	24/76	2.43	27.80±0.04	27.20±0.04	27.70±0.04	27.80±0.05	56.00	60.00	60.00	60.00
E2	+1	-1	46/54	2.43	17.50±0.04	17.30±0.08	18.20±0.13	17.40±0.08	12.00	36.00	40.00	40.00
E3	-1	+1	24/76	10.37	30.40±0.24	30.80±0.04	31.70±0.11	29.20±0.11	25.00	41.66	45.83	45.83
E4	+1	+1	46/54	10.37	8.67±0.22	8.91±0.30	7.87±0.32	7.84±0.37	0.00	0.00	7.41	11.11
E5	-1.41	0	20/80	6.40	36.60±0.23	37.70±0.25	39.10±0.19	39.80±0.16	50.00	56.52	60.87	60.87
E6	+1.41	0	50/50	6.40	10.00±0.17	9.74±0.24	9.60±0.23	9.10±0.27	0.00	8.00	14.00	16.00
E7	0	-1.41	35/65	0.80	19.90±0.04	20.10±0.08	19.50±0.04	20.70±0.09	58.33	62.50	62.50	62.50
E8	0	+1.41	35/65	12.00	20.60±0.19	20.30±0.15	20.40±0.11	20.30±0.09	8.00	18.00	24.00	28.00
E9	0	0	35/65	6.40	28.60±0.16	29.40±0.08	30.40±0.04	28.40±0.08	16.67	37.50	37.50	37.50
E10	0	0	35/65	6.40	28.40±0.10	27.90±0.07	28.80±0.04	28.50±0.04	16.67	37.50	37.50	37.50
E11	0	0	35/65	6.40	28.30±0.07	29.30±0.48	28.40±0.05	28.40±0.05	16.67	36.00	36.00	40.00

Table S2: Morphology of the 11 samples from the CCDR 22 experimental runs. The Span values for t1 and t30 are represented in bold in the upper left side of the images.

	t1	t7	t15	t30
**E1**	**1.502** 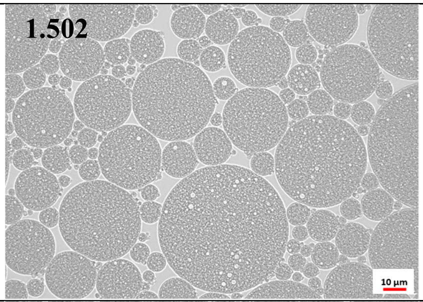	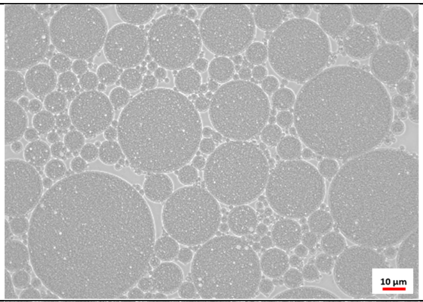	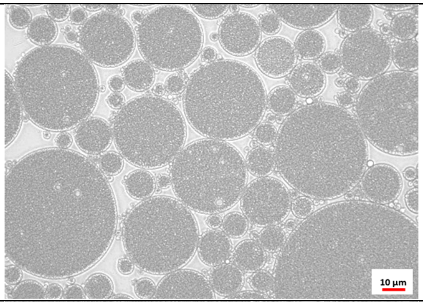	**1.453** 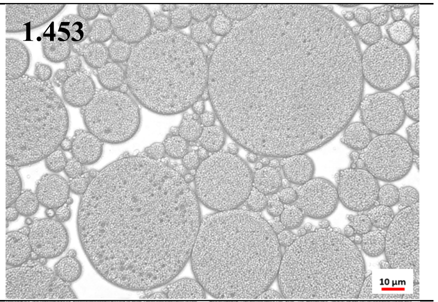
**E2**	**1.334** 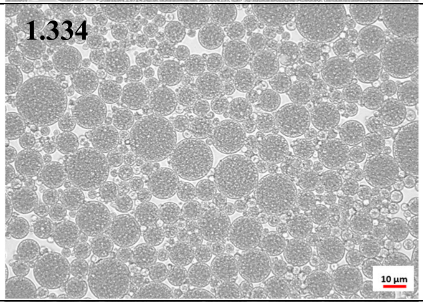	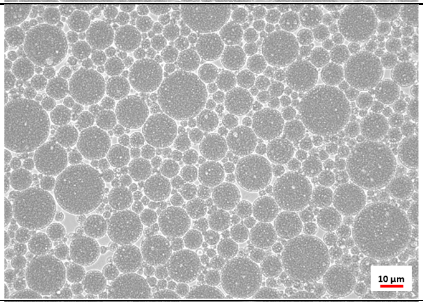	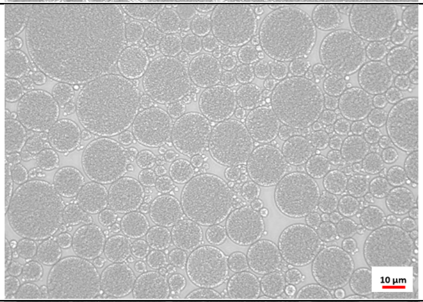	**1.337** 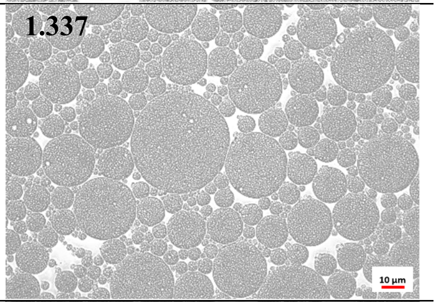
**E3**	**1.453** 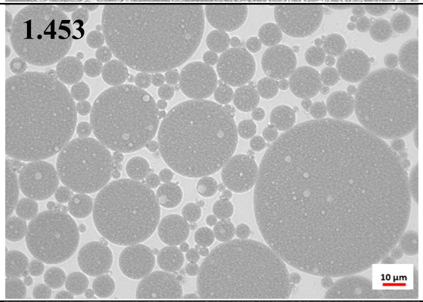	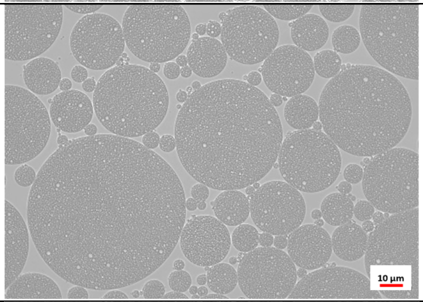	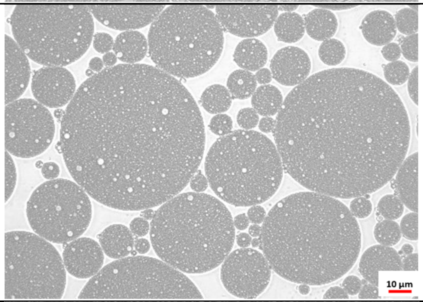	**1.629** 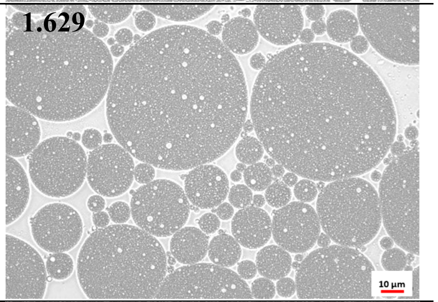
**E4**	**0.621** 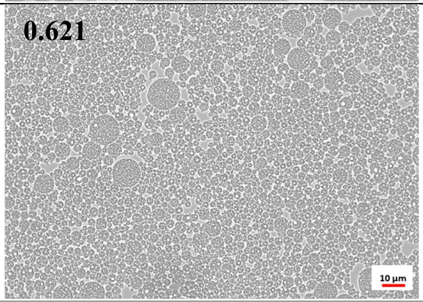	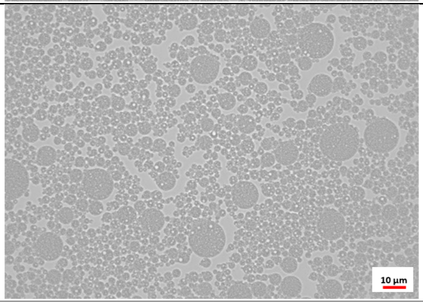	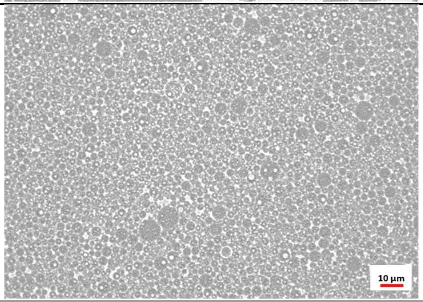	**0.495** 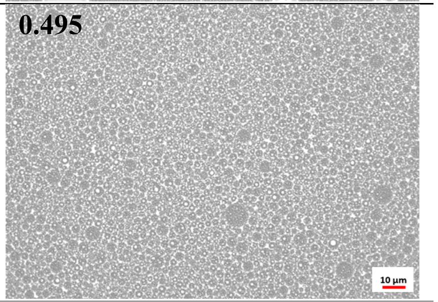

E5

E6

E7

E8

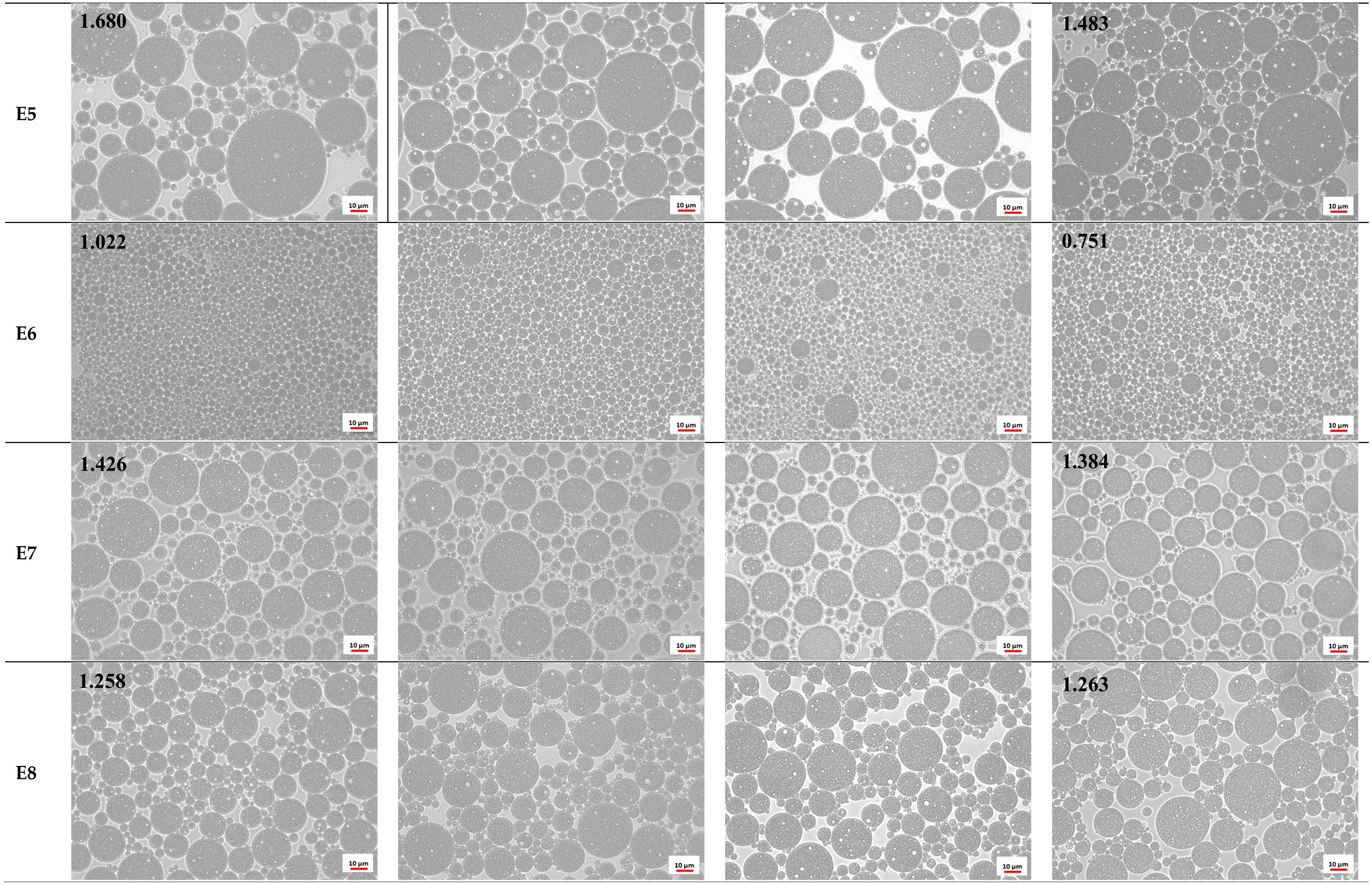

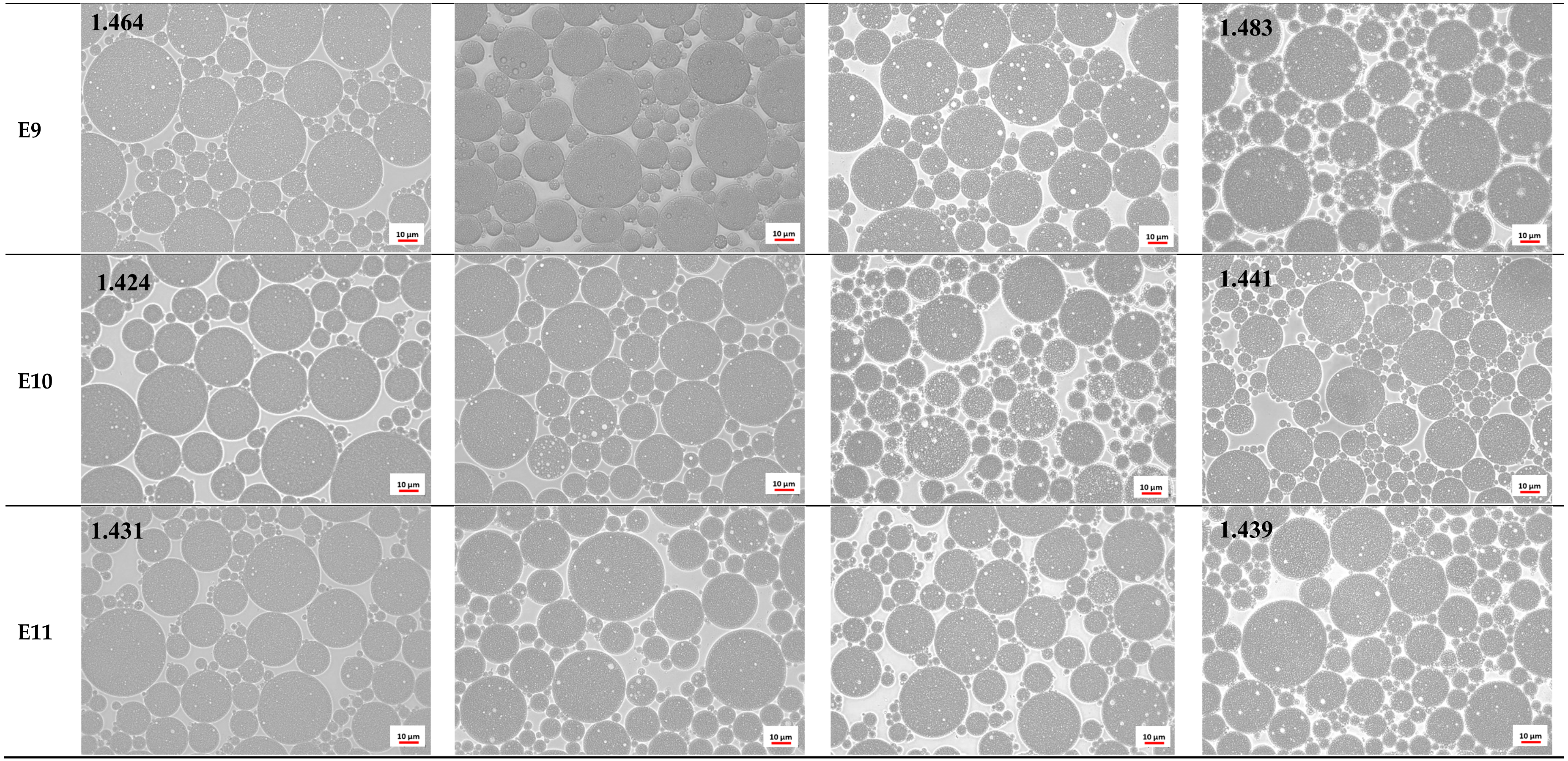
E9
1.464
1.483
E10
1.424
1.441
E11
1.431
1.439
10 µm

Table S3: Design matrix of the Central Composite Rotatable Design (CCRD) with independent variables emulsion ratio (v/v) and colorant concentration (%) and the results for responses: Colorimetric parameters: a* and L* for the 1, 7, 15 and 30 days after the emulsion production.

Run	Independent Variables				Dependent Variables (Colorimetric parameters)											
	coded levels		real values		t1	t7	t15	t30	t1	t7	t15	t30	t1	t7	t15	t30
	x1	x2	Emulsion ratio ((W_1/O)/W_2) (v/v)	Colorant concentration (%)	*a**				*L**				*b**			
E1	-1	-1	24/76	2.43	21.31±0.07	20.68±0.02	19.53±0.07	18.10±0.03	56.24±0.02	56.62±0.08	56.65±0.04	57.86±0.04	-1.26±0.02	-1.40±0.02	-1.77±0.04	-2.84±0.04
E2	+1	-1	46/54	2.43	21.75±0.06	21.54±0.02	21.31±0.14	20.92±0.04	55.19±0.07	55.49±0.03	55.50±0.19	56.64±0.02	-1.23±0.08	-1.24±0.02	-1.42±0.07	-1.57±0.01
E3	-1	+1	24/76	10.37	27.98±0.08	27.51±0.08	27.11±0.03	27.04±0.01	45.17±0.14	45.70±0.06	45.55±0.03	46.93±0.01	0.61±0.04	0.15±0.05	-0.08±0.04	-0.40±0.01
E4	+1	+1	46/54	10.37	28.92±0.07	28.96±0.04	28.98±0.02	28.98±0.01	41.44±0.05	41.69±0.06	41.72±0.01	41.95±0.02	0.87±0.02	1.01±0.02	0.97±0.01	0.81±0.01
E5	-1.41	0	20/80	6.40	26.91±0.05	26.13±0.12	25.39±0.06	24.43±0.01	48.09±0.04	48.26±0.05	48.45±0.06	48.52±0.02	-0.19±0.01	-0.60±0.07	-1.07±0.02	-1.89±0.03
E6	+1.41	0	50/50	6.40	27.79±0.01	27.65±0.06	27.94±0.16	27.61±0.06	44.52±0.02	44.64±0.04	44.83±0.12	45.20±0.03	-0.11±0.01	0.05±0.01	-0.07±0.04	-0.51±0.05
E7	0	-1.41	35/65	0.80	17.09±0.06	16.45±0.12	15.71±0.02	14.17±0.05	62.01±0.04	62.13±0.04	62.28±0.02	63.07±0.13	-1.12±0.02	-1.03±0.02	-1.24±0.03	-1.44±0.09
E8	0	+1.41	35/65	12.00	28.16±0.04	27.99±0.03	27.97±0.01	27.85±0.06	42.19±0.08	42.42±0.10	42.43±0.08	42.48±0.05	0.53±0.02	0.40±0.00	0.31±0.03	0.12±0.02
E9	0	0	35/65	6.40	26.97±0.04	26.56±0.09	26.44±0.03	25.89±0.03	46.52±0.12	46.73±0.14	46.77±0.03	46.81±0.03	-0.19±0.01	-0.52±0.01	-0.63±0.02	-1.06±0.03
E10	0	0	35/65	6.40	27.01±0.05	26.60±0.05	26.46±0.02	26.07±0.02	46.40±0.03	46.66±0.11	46.80±0.09	46.88±0.05	-0.15±0.03	-0.51±0.02	-0.61±0.03	-0.86±0.02
E11	0	0	35/65	6.40	21.31±0.07	20.68±0.02	19.53±0.07	18.10±0.03	56.24±0.02	56.62±0.08	56.65±0.04	57.86±0.04	-1.26±0.02	-1.40±0.02	-1.77±0.04	-2.84±0.04

Table S4: The experimental values of the validation formulations. V1: 48/52 (W_1/O)/W_2 ratio (v/v) and 6 wt% of colorant concentration. V2: 41/59 (W_1/O)/W_2 ratio (v/v) and a colourant concentration of 11 wt.%.

**Time**	**$D_{4,3}$**		**CI**		***L****		***a****	
	**V1**	**V2**	**V1**	**V2**	**V1**	**V2**	**V1**	**V2**
t1	9.72±0.19	17.63±0.25	0±0.001	0±0.001	43.80±0.01	37.32±0.05	26.13±0.02	26.81±0.02
t7	9.76±0.05	17.43±0.38	6.32±0.94	6.17±2.14	44.04±0.06	37.24±0.02	26.23±0.01	26.80±0.02
t15	9.68±0.1	18.03±0.52	7.60±0.16	7.85±1.22	44.04±0.01	37.47±0.02	25.88±0.01	26.50±0.01
t30	9.62±0.1	18.25±0.67	14.55±0.99	17.28±1.07	44.11±0.03	37.55±0.01	25.79±0.01	26.34±0.01

Table S5: Regression parameters (a's coefficients of equation 1) for the volume- mean droplet size (μm), creaming index (%) and colorimetric parameters (*a** and *L**) responses, and their second-order models after seven days (t7).

Sorce	Droplet size ($D_{4,3}$)		Creaming Index (CI)		*L**		*a**	
	**a's coefficients# (actual factors)**	***p*-Value**	**a's coefficients# (actual factors)**	***p*-Value**	**a's coefficients# (actual factors)**	***p*-Value**	**a's coefficients# (actual factors)**	***p*-Value**
Model	--	0.0002	--	<0.0001	--	<0.0001	--	<0.0001
Intercept	+2.6	<0.0001	+60.4	<0.0001	+65.3	<0.0001	+13.1	<0.0001
x_1- Emulsion Ratio	+1.25	<0.0001	+0.96	<0.0001	–0.01	0.0005	+0.05	0.0025
x_2- Colorant Concentration	+5.96	0.3812	–0.15	<0.0001	–3.45	<0.0001	+2.75	<0.0001
x_1x_2	–0.0686	0.0154	–0.1011	0.0056	–0.0165	0.0380	--	n.s.
$(x_1)^2$	–0.0231	0.0102	–0.0262	0.0093	--	n.s.	--	n.s.
$(x_2)^2$	–0.2893	0.0013	--	n.s.	+0.185	<0.0001	–0.140	<0.0001
Quality parameter	Values		Values		Values		Values	
Adequate precision	21.90		44.59		56.86		67.31	
R^2	0.9832		0.9937		0.9958		0.9944	
R^2_{adj}	0.9663		0.9894		0.9929		0.9920	
R^2_{pred}	0.8887		0.9769		0.9740		0.9794	

#Parameter not statistically significant (n.s.; *p*-Value≥0.10): not included in the model since it did not affect its hierarchy.

Table S6: Regression parameters (a's coefficients of equation 1) for the volume- mean droplet size (μm), creaming index (%) and colorimetric parameters (*a** and *L**) responses, and their second-order models after fifteen days (t15).

Sorce	Droplet size ($D_{4,3}$)		Creaming Index (CI)		*L**		*a**	
	**a's coefficients[#] (actual factors)**	***p*-Value**	**a's coefficients[#] (actual factors)**	***p*-Value**	**a's coefficients[#] (actual factors)**	***p*-Value**	**a's coefficients[#] (actual factors)**	***p*-Value**
Model	--	0.0003	--	<0.0001	--	<0.0001	--	<0.0001
Intercept	+2.6	<0.0001	+90.11	<0.0001	+65.5	<0.0001	+11.0	<0.0001
x_1- Emulsion Ratio	+1.19	<0.0001	−0.74	<0.0001	−0.02	0.0004	+0.08	<0.0001
x_2- Colorant Concentration	+6.63	0.4066	−1.69	<0.0001	−3.47	<0.0001	+2.93	<0.0001
x_1x_2	−0.0820	0.0151	−0.1055	0.0042	−0.0153	0.0389	--	n.s.
$(x_1)^2$	−0.0217	0.0253	--	n.s	--	n.s.	--	n.s.
$(x_2)^2$	−0.3059	0.0021	+0.1715	0.0170.	+0.183	<0.0001	−0.149	<0.0001
Quality parameter	Values		Values		Values		Values	
Adequate precision	19.93		40.76		60.63		73.85	
R^2	0.9788		0.9925		0.9963		0.9965	
R^2_{adj}	0.9576		0.9875		0.9938		0.9950	
R^2_{pred}	0.8609		0.9597		0.9769		0.9855	

[#]Parameter not statistically significant (n.s.; *p*-Value≥0.10): not included in the model since it did not affect its hierarchy.

Table S7: Regression parameters (a's coefficients of equation 1) for the volume- mean droplet size (µm), creaming index (%) and colorimetric parameters (*a** and *L**) responses, and their second-order models after third days (t30).

Sorce	Droplet size ($D_{4,3}$)		Creaming Index (CI)		*L**		*a**	
	**a's coefficients[#] (actual factors)**	***p*-Value**	**a's coefficients[#] (actual factors)**	***p*-Value**	**a's coefficients[#] (actual factors)**	***p*-Value**	**a's coefficients[#] (actual factors)**	***p*-Value**
Model	--	0.0009	--	<0.0001	--	<0.0001	--	<0.0001
Intercept	+48.10	<0.0001	+92.15	<0.0001	+65.8	<0.0001	+8.66	<0.0001
x_1- Emulsion Ratio	+0.85	0.0002	−0.80	<0.0001	+0.01	0.0041	+0.10	<0.0001
x_2- Colorant Concentration	+2.74	0.4324	−2.36	<0.0001	−3.53	<0.0001	+3.15	<0.0001
x_1x_2	n.s	n.s.	−0.0843	0.0206	−0.0215	0.0703	--	n.s.
$(x_1)^2$	n.s.	n.s.	--	n.s	--	n.s.	--	n.s.
$(x_2)^2$	−0.2359	0.0412	+0.1893	0.0200	+0.2005	<0.0001	−0.1567	<0.0001
Quality parameter	Values		Values		Values		Values	
Adequate precision	12.31		32.99		38.08		62.03	
R^2	0.8930		0.9886		0.9904		0.9950	
R^2_{adj}	0.8471		0.9811		0.9840		0.9929	
R^2_{pred}	0.7054		0.9428		0.9433		0.9812	

[#]Parameter not statistically significant (n.s.; *p*-Value≥0.10): not included in the model since it did not affect its hierarchy.

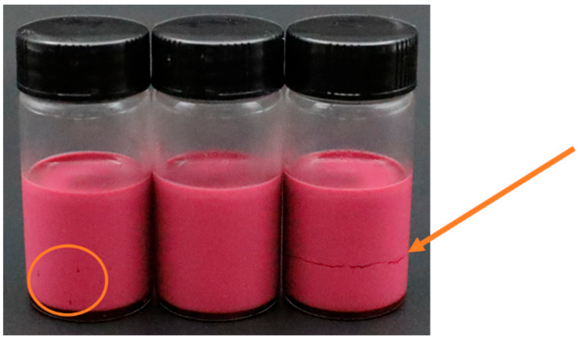

Figure S1. The visual inspection of V2, where the fraction of colorant is in the outer phase of the emulsion

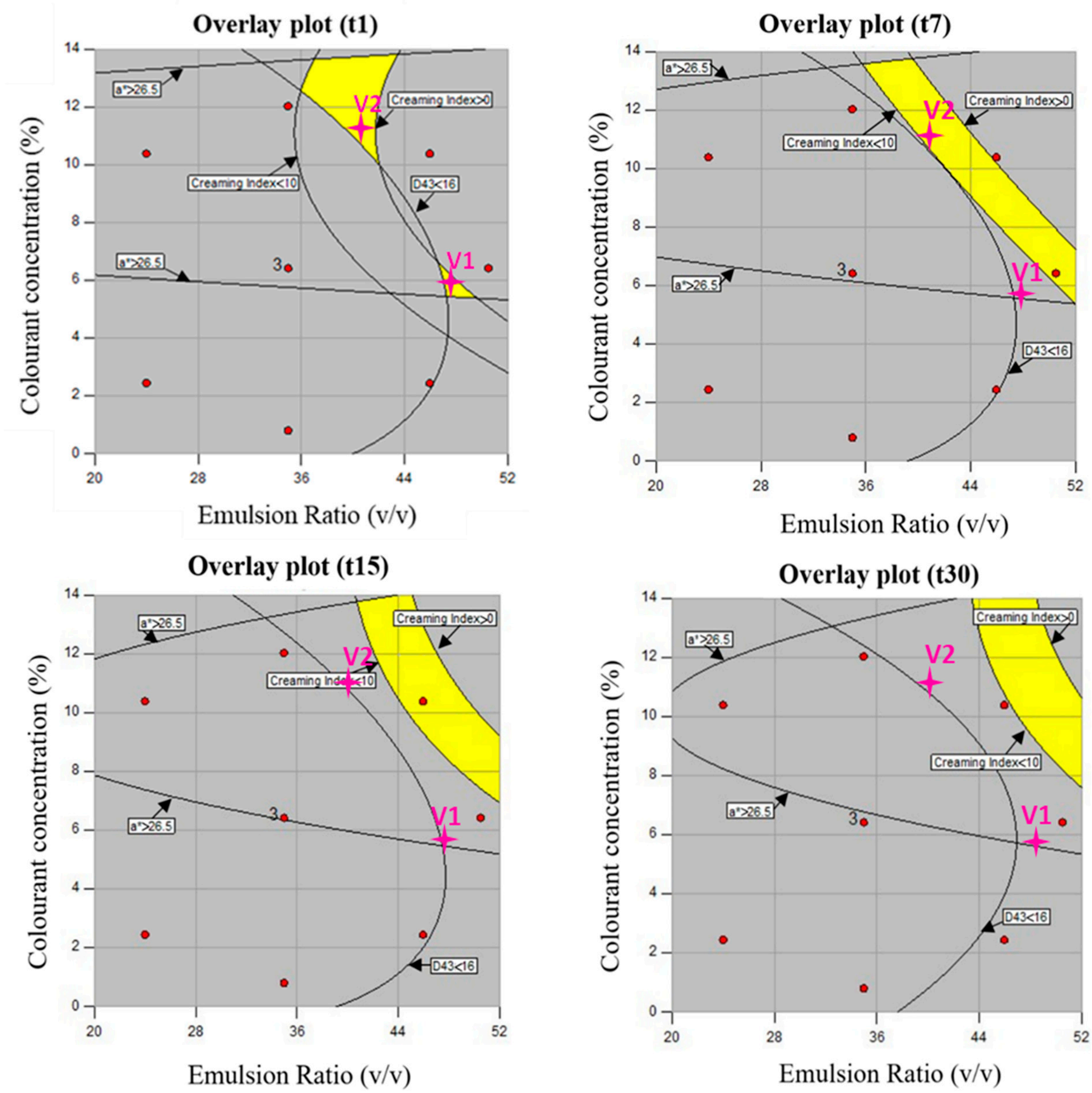

Figure S2. The overlay plot for t1, t7, t15 and t30 after double emulsion production.
